# Supplementary material for: Process evaluation of an interorganizational cooperation initiative in vocational rehabilitation: the Dirigo project
Source: BMC Public Health. 2017 May 11;17:431. doi: 10.1186/s12889-017-4357-x (PMC5426082; doi:10.1186/s12889-017-4357-x)
Supplement: Additional file 1: — Guides for interviews and focus groups. (ZIP 240 kb) [file 12889_2017_4357_MOESM1_ESM.zip › 2012 guide for fall focus groups with staffR3.docx]

# Focus groups, staff, fall 2012

The theme for the focus groups is workplace learning and cooperation within the project, focusing on how the project works with supporting and developing the common work in a continuing learning process.

The following questions will guide the discussion:

- In what way do you think that the content of the training has been put into practice?
  - In a structured way, or up to each person?
- In what ways does your work in the project differ from how you worked before?
  - What do you know today that you did not know before?
  - What do you do differently? Why? How?
- Give an example of a situation where your competence has been challenged.
  - How did you manage this situation?
- In what ways do you work, on a workplace level, with feedback?
  - What support is available and how is it used?
  - How are conflicts or problems managed?
  - Is creativity and new ideas promoted?
- Is there anything in the project that needs to be done differently, regarding methods or procedures?
