# Supplementary material for: Process evaluation of an interorganizational cooperation initiative in vocational rehabilitation: the Dirigo project
Source: BMC Public Health. 2017 May 11;17:431. doi: 10.1186/s12889-017-4357-x (PMC5426082; doi:10.1186/s12889-017-4357-x)
Supplement: Additional file 1: — Guides for interviews and focus groups. (ZIP 240 kb) [file 12889_2017_4357_MOESM1_ESM.zip › 2012 guide for focus group with managersR3.docx]

# Focus group, managers, 2012

The aim of the focus group is to explore the manager group’s background, previous experiences and expectations of the project. It is based on the following questions:

- What has happened so far? Where is the project today?
- Education and background, previous experience of managing cooperative projects?
- Expectations of the project, what will it lead to?
- What practical conditions are in place, and are there hinders for reaching a purposeful cooperation within the project?
  - Regulations
  - Organizational isssues
  - Personal attitudes
  - Competences
- How will the project manage potential hinders?
- What knowledge about the target groups exists in the managerial group?
- What values drives the work in the project? Relationship to regular practice in the participating organizations?
  - Who is responsible for promoting change? Participation of clients?
- How will the project manage issues of equal treatment and equality?
- What competences does the managers need?
- Implementation plans
