# Supplementary material for: Process evaluation of an interorganizational cooperation initiative in vocational rehabilitation: the Dirigo project
Source: BMC Public Health. 2017 May 11;17:431. doi: 10.1186/s12889-017-4357-x (PMC5426082; doi:10.1186/s12889-017-4357-x)
Supplement: Additional file 1: — Guides for interviews and focus groups. (ZIP 240 kb) [file 12889_2017_4357_MOESM1_ESM.zip › 2012 guide for spring focus groups with staffR3.docx]

# Focus groups, staff, spring 2012

The aim if the focus groups is to explore the officials’ existing knowledge, experiences and expectations before the project starts. The following questions guide the discussions:

- What has happened so far?
- Earlier experience of cooperative work?
- What practical conditions or hinders exists today for a purposeful cooperation between authorities?
  - Regulations
  - Organizational
  - Personal attitudes
  - Competences
- What knowledge does the staff have about the target groups?
- Will the work be carried out differently for different groups?
  - Different labor market possibilities
- What values drives the work within the authorities involved?
  - Whose responsibility to promote change?
  - Participation?
- Expectations and views on the project and the proposed methods
  - Supported Employment
  - Motivational Interviewing
  - Values
- How does the work forms in the project differ from how you have been working before?
- Do you have further ideas of how the methods could be developed?
- Is there a need for competence development/training in any issues?
