# Supplementary material for: Process evaluation of an interorganizational cooperation initiative in vocational rehabilitation: the Dirigo project
Source: BMC Public Health. 2017 May 11;17:431. doi: 10.1186/s12889-017-4357-x (PMC5426082; doi:10.1186/s12889-017-4357-x)
Supplement: Additional file 1: — Guides for interviews and focus groups. (ZIP 240 kb) [file 12889_2017_4357_MOESM1_ESM.zip › 2012 interview guide for departing managerR3.docx]

# Interview guide, departing manager, 2012

- What do you do today?
- What lead up to the departure?
  - How was the process?
  - What made you make the decision?
  - How did you feel before making the decision?
  - How do you feel today?
- What’s your current perspective on the project?
  - Pros and cons?
  - What did not work (examples)?
  - Why not?
  - Whose responsibility?
  - How did the management work? The staff?
  - What did you do to manage the things that did not work?
  - What could you have done differently?
  - How do you perceive the goals and strategies of the project?
- What would you have changed if the project was to start today?
- What would it have taken for you to stay?
