# Supplementary material for: Process evaluation of an interorganizational cooperation initiative in vocational rehabilitation: the Dirigo project
Source: BMC Public Health. 2017 May 11;17:431. doi: 10.1186/s12889-017-4357-x (PMC5426082; doi:10.1186/s12889-017-4357-x)
Supplement: Additional file 1: — Guides for interviews and focus groups. (ZIP 240 kb) [file 12889_2017_4357_MOESM1_ESM.zip › 2012 interview guide for managersR3.docx]

# Interview guide, managers, 2012

The aim of the interview is to focus on how the process has been perceived, and on future development needs and goals.

- Describe your role in the project. Responsibilities? Amount of time in your position?
- How do you think the project has developed?
  - Start-up
  - Where is the project today?
  - What has/has not worked? (Give examples)
  - Why? Whose responsibility?
- What would you change if the project started today?
  - Does the project do things today you did not think from the beginning?
- How does the project organization work? Relationships and communication managers-staff?
- How do you perceive the project’s goals and strategies?
  - What do you think the long-term effects will be?
  - Strategic influence, what parts can be implemented into your regular organization? How does the organization prepare for this? Have results been fed back to your organization? How?
- How does the cooperation between participating organizations work?
