# Supplementary material for: Process evaluation of an interorganizational cooperation initiative in vocational rehabilitation: the Dirigo project
Source: BMC Public Health. 2017 May 11;17:431. doi: 10.1186/s12889-017-4357-x (PMC5426082; doi:10.1186/s12889-017-4357-x)
Supplement: Additional file 1: — Guides for interviews and focus groups. (ZIP 240 kb) [file 12889_2017_4357_MOESM1_ESM.zip › 2013 guide for fall focus groups with staffR3.docx]

# Focus groups, staff, spring 2013

The starting point is to follow up the results from the latest evaluation report, which focused on the views from staff and management on the development of the project, communication, goals and methods. We will focus on the following issues:

- Management and communication. Staff want more clarity and support in methodological development, and more feedback.
  - How can we make sure that this is achieved?
  - How can the staff support each other in applying methods (e.g., MI)?
- Conflicts between production and development. Much focus on production based on how the project was designed, risk that development will not be prioritized.
  - How ca these goals be combined?
  - Responsibilities of managers and staff?
- Value work is done informally, and staff has trouble seeing it.
  - How can this become more visible?
  - How to strengthen dialogue within the work groups?
  - Managers’ and staff responsibilities in this?
- Communication between offices is limited, risking to result in different project developments.
  - How can this be improved?
  - To what extent do you work differently in the different offices?
  - Is methods applied differently?
  - How can a common development be facilitated?

Since last time, changes has taken place in the managerial level. We will also discuss if these changes have affected the work in the project. The focus groups will mix participants from the different offices.
