# Supplementary material for: Process evaluation of an interorganizational cooperation initiative in vocational rehabilitation: the Dirigo project
Source: BMC Public Health. 2017 May 11;17:431. doi: 10.1186/s12889-017-4357-x (PMC5426082; doi:10.1186/s12889-017-4357-x)
Supplement: Additional file 1: — Guides for interviews and focus groups. (ZIP 240 kb) [file 12889_2017_4357_MOESM1_ESM.zip › 2013 guide for spring focus groups with staffR3.docx]

# Focus groups, staff, 2013

In november, we will arrange focus groups related to methodological issues in the project offices. The aim is to discuss cases, how you have worked in them, and what results you reached. Before this occasion, please (individually or in groups of two) choose two anonymized cases:

- One where you think that you reached good results and where you could apply the different methods that are unique to the project
- One where you struggled with reaching the desired results, or where methods could not be applied as planned

The cases you choose should be documented in a way so that we can understand the methods or activities that were used.
