# Supplementary material for: Process evaluation of an interorganizational cooperation initiative in vocational rehabilitation: the Dirigo project
Source: BMC Public Health. 2017 May 11;17:431. doi: 10.1186/s12889-017-4357-x (PMC5426082; doi:10.1186/s12889-017-4357-x)
Supplement: Additional file 1: — Guides for interviews and focus groups. (ZIP 240 kb) [file 12889_2017_4357_MOESM1_ESM.zip › 2013 interview guide for managersR3.docx]

**Interview guide, managers, 2013**

- The target group you are working with in the project, is it the one you imagined before the project started?
- The methods you are using, how did you decide on them?
- What is your job in the project, in relation to the staff? To the steering group?
- How do you support innovative thinking in the project?
- How do you document the work in the project? Do you specifically document what works? How is the work organized?
- How do you work with developing the methods?
- What problems are the project designed to solve?
- There have been some changes in the project. How has these changes affected the project and how it operates?
  - Has it affected the work group?
- How do you work with employers?
- There is a transnational element to the project, how is this done?
- Is there anything else you would like to discuss?
