# Supplementary material for: Process evaluation of an interorganizational cooperation initiative in vocational rehabilitation: the Dirigo project
Source: BMC Public Health. 2017 May 11;17:431. doi: 10.1186/s12889-017-4357-x (PMC5426082; doi:10.1186/s12889-017-4357-x)
Supplement: Additional file 1: — Guides for interviews and focus groups. (ZIP 240 kb) [file 12889_2017_4357_MOESM1_ESM.zip › 2014 interview guide for fundersR3.docx]

**Interview guide, funding agency, 2014**

- What has been your role during the project?
- What has been your strategy?
- Who did you have contacts with?
- What is your perspective on how the project has developed?
- Why did the project have start-up problems?
- Have you followed up the results and effects of the project?
- How do you perceive the project’s results? What did it lead to?
- The goals changed during the project. What is your perspective on that?
- The project also changed, reducing from three to two offices, for example. Why was that?
- Have you as a funding agency had any influence over how the project developed?
- Have you communicated with the project about the closing?
- Are you participating in discussions about implementation of project methods into regular work? Do you have any opinions about this?
- Are there, in your perspective, any specific experiences from the project that you think would be possible to develop further?
- In general, how do you think the contacts with the project has worked?
- In general, do you think this has been a good project? Has it lead to new knowledge?
- Is there anything else you would like to raise?
