# Supplementary material for: Process evaluation of an interorganizational cooperation initiative in vocational rehabilitation: the Dirigo project
Source: BMC Public Health. 2017 May 11;17:431. doi: 10.1186/s12889-017-4357-x (PMC5426082; doi:10.1186/s12889-017-4357-x)
Supplement: Additional file 1: — Guides for interviews and focus groups. (ZIP 240 kb) [file 12889_2017_4357_MOESM1_ESM.zip › 2014 interview guide for staffR3.docx]

# Interview guide, staff, spring 2014

Retrospective

- Your professional development
  - Changes in how you meet clients?
  - Changes in how you approach regulations?
- The project’s development
  - Balance between methodological development and production?
  - How was the methods applied?
  - Use of measures in the regular organizations?
  - Development for participants – do you consider the project to have been effective?
- Transnational activities
  - Methodological aspects of the trips that was carried out?
- Support from managers and colleagues in applying methods
  - How did you use existing resources in the organizations for support?
    - Difference compared to prior to the project?
  - How would you characterize the management in the project (transformative, transactional, laissez-faire)?

The project closing

- In what way have you prepared for the closing of the project?
  - Cases?
  - Transition to what type of measures?
- In what way did the management prepare the closing?
  - Routines for documentation?
- How will you use your experience from the project in the future?
  - What will you do next?
- What will be implemented?
  - Relationship between the project and the regular organization?
