# Supplementary material for: Process evaluation of an interorganizational cooperation initiative in vocational rehabilitation: the Dirigo project
Source: BMC Public Health. 2017 May 11;17:431. doi: 10.1186/s12889-017-4357-x (PMC5426082; doi:10.1186/s12889-017-4357-x)
Supplement: Additional file 1: — Guides for interviews and focus groups. (ZIP 240 kb) [file 12889_2017_4357_MOESM1_ESM.zip › Dirigo process article BMC revised again 170424R3.docx]

**Process evaluation of an interorganizational cooperation initiative in vocational rehabilitation: The Dirigo project**

Christian Ståhl^1^, Åsa Andersén^2^, Ingrid Anderzén^2^, Kjerstin Larsson^2,3^

1) National Centre for Work and Rehabilitation, Department of Medical and Health Sciences, Linköping University, Sweden

2) Department of Public Health and Caring Sciences, Uppsala University, Sweden

3) Department of Neurobiology, Care Sciences and Society, Section of Social Work, Karolinska Institutet, Sweden

Christian Ståhl (corresponding author)

National Centre for Work and Rehabilitation

Department of Medical and Health Sciences

Linköping University

583 81 Linköping

Sweden

Telephone: +46 (0)13-28 26 90

E-mail: christian.stahl@liu.se

Åsa Andersén

Department of Public Health and Caring Sciences

Uppsala University

Box 564

751 22 Uppsala

Sweden

Telephone: +46 (0) 18-471 65 75

E-mail: asa.andersen@pubcare.uu.se

Ingrid Anderzén

Department of Public Health and Caring Sciences

Uppsala University

Box 564

751 22 Uppsala

Sweden

Telephone: +46 (0) 18-471 65 75

E-mail: ingrid.anderzen@pubcare.uu.se

Kjerstin Larsson

Department of Public Health and Caring Sciences

Uppsala University

Box 564

751 22 Uppsala

Sweden

Telephone: +46 (0) 18-471 66 35

E-mail: kjerstin.larsson@pubcare.uu.se

**Abstract**

*Background:* This study analyzes the process of establishing and developing a cooperative vocational rehabilitation project with special focus on organizational and professional aspects. In the project, officials from the Swedish Social Insurance Agency and the Swedish Public Employment Service worked cooperatively with participants on long-term sick leave, youths with disability benefits, and people receiving social allowances. The officials used Motivational Interviewing (MI) as a method when meeting participants, and were able to offer flexible and tailored case management. The goal was to improve work ability and promote self-sufficiency.

*Methods:* The process evaluation was carried out through continuous data collection throughout the project (2012-2014), resulting in a total of 28 individual interviews and 17 focus groups with officials and managers. The material was categorized through an inductive content analysis, and analyzed using social capital as a theoretical frame.

*Results:* The evaluation points to how issues related to design, organization and management contributed to the project not reaching its goals, e.g. problems with recruitment of participants, the funding structure, and staffing problems on the managerial level. Still, officials reported positive effects of close cooperation, which was perceived as facilitating the case management by fostering a mutual understanding and access to resources and rehabilitation measures from more than one authority.

*Conclusions:* Cooperative work combined with the use of MI and flexible case management seem to promote an increased trust between officials from different authorities and participants, which in the study is conceptualized as bonding and bridging social capital (between officials) and linking social capital (between officials and participants). The organizational problems combined with the relatively large differences in approaches between the project and regular practice obstructed implementation, where the authorities involved did not appear to be ready for implementing methodologies that require organizational restructuring.

*Keywords:* Vocational Rehabilitation; Cooperation; Employment; Motivational Interviewing; Social Capital; Sweden

**Background**

Cooperation between stakeholders in vocational rehabilitation has been advised in many studies, often based on the recognition that differing stakeholder perceptions and system-related communication problems may hinder a purposeful rehabilitation process. The evidence for the effectiveness of cooperative strategies is still mixed, where some studies show little (or even negative) effects on rehabilitation outcomes [[1](#_ENREF_1), [2](#_ENREF_2)], while others show more positive results [[3-6](#_ENREF_3)]. Professionals working in cooperation projects often experience them as leading to tighter and more constructive cooperation [[1](#_ENREF_1), [7](#_ENREF_7)]. These divergent results are to a large extent a result of variation in the design of cooperative interventions, which may include different stakeholders in different ways, and that they are conducted in different jurisdictions with different target groups. The results of cooperation further depend on factors such as which organizations that participate, the allocated resources, in what context cooperation takes place, and whether managers and staff are committed to the cooperation or not. What is often stated in research, however, is that interorganizational cooperation generally demands a considerable amount of initial resources, that it takes time to develop purposeful cooperation structures and that the socioeconomic effects will not be immediate [[1](#_ENREF_1), [7](#_ENREF_7), [8](#_ENREF_8)]. These factors make the effects even harder to study.

The large variety in design of cooperation initiatives calls for a close attention to both sufficient detail in descriptions of interventions, and to a proper contextualization of studies. One way of achieving such detail and context is to conduct process evaluations alongside studies of intervention outcomes. In this study, we present a process evaluation of a cooperation project (Dirigo) between Swedish state authorities in rehabilitation, targeting people on long-term sick leave, and young people on disability benefits.

The aim of this article is to analyze the process of establishing and developing a cooperative vocational rehabilitation project. A special focus was placed on the organizational and professional aspects, where specific research questions are:

- What are the experiences of the officials involved with the cooperative methodology in the project?
- What are the experiences of the officials and managers involved with the establishment and management of the project?

The study adds to the existing literature by offering a process perspective on establishing cooperative work forms, both regarding how such work is perceived by professionals, and the challenges of implementation.

***The Dirigo project***

Dirigo was a project that ran from January 2012 to April 2014. It was based on close cooperation between the Swedish Sickness Insurance Agency (SSIA) and the Swedish Public Employment Services (SPES), where officials from the two authorities worked in pairs with participants. The project goals were to improve participants’ work ability, and to promote self-sufficiency, work or studies. The staff was located in shared workspaces in three dedicated offices located in Stockholm, Sweden, with one project leader per office. Halfway through the project, two offices were combined into one due to changes in the amount of participants, and changes in staffing. The project was co-financed by the European Social Fund (ESF) and the participating organizations, and had a steering group consisting of managers from the SSIA and the SPES, alongside municipal managers.

The target groups were people on long-term sick leave (>180 days, most participants had been sick-listed for several years), and people on youth disability benefit (a specific benefit given to people up to 30 years of age if assessed as permanently work disabled). There was also a third group, people on social allowances (means-tested municipal benefits to citizens who cannot provide for themselves through other sources), where municipal social workers were to work with these together with officials from other authorities. However, only one social worker was recruited and this group of participants was therefore smaller. In the evaluation, this part of the project was given less focus since it appeared more peripheral, and since the evaluation would be focusing on the efforts of only one professional.

In total, the project had 629 participants. For a description of the group and the success rate, see Table 1 (these figures were delivered by the project management at the closure of the project; unfortunately, they do not allow for statistical analyses, and no figures are available about the sustainability of the results). Success indicates that the participant is working, studying or is actively looking for a job (i.e., enrolled in activities within the SPES with unemployment benefits, and no longer on sickness or disability benefits). The project goals were not met for the main target groups; however, the success rate for these groups was comparable to the results of the regular cooperative structure between the SSIA and the SPES [[9](#_ENREF_9)].

Table 1: Project participants, goals and success rate (according to the project’s final report).

| **Group** | **Participants** | **Project goals** | **Success rate** |
| --- | --- | --- | --- |
| Long term sick leave | 235 | 70-85% should be self-sufficient | 38% |
| Youth disability benefit | 353 | >30 % should be self-sufficient | 21% |
| Social allowances | 41 | 20-40% should be self-sufficient | 43% |

In the Swedish system, the SSIA is responsible for administering sickness insurance and for coordinating the rehabilitation process. The medical rehabilitation is generally performed in local primary health care centers or hospitals, while the occupational rehabilitation is the responsibility of the employers. The SPES is responsible for matching job seekers with employers, but also for providing aid in vocational rehabilitation. This is primarily offered to unemployed with functional disabilities.

The intervention offered in the project differed significantly from regular practice, where officials from the different authorities only cooperate with professionals from other organizations at specific time points. In regular practice, the target groups for the project do not generally have contact with the SPES, since they are managed by the SSIA while receiving sickness or disability benefits. The officials in the projects also had relatively few cases; the caseload was around 30-40 cases per official, compared to over 100 in regular practice. This allowed for more time with the participants, and more flexibility in service delivery. Officials were also trained in Motivational Interviewing (MI) as a guiding tool for meetings with the participants. MI is a method for promoting change that has been evaluated with convincing results in several areas, e.g. smoking cessation and drug rehabilitation [[10](#_ENREF_10), [11](#_ENREF_11)]. The intervention resembled a tailored case management model, where officials from two authorities worked in pairs in close cooperation with the participant in supporting their rehabilitation process and contacts with prospective employers.

**Methods**

The evaluation of the Dirigo project had a mixed methods approach with the aim to analyze both the results of the intervention and the process of establishing and developing the project. The process evaluation, which is presented in this article, focused on the organizational and professional aspects of the project, through continuous interviews and focus groups with officials and managers. The evaluation was designed according to the principles of interactive research, where the evaluation takes place continuously during the project [[12](#_ENREF_12)]. Feedback reports were written twice every year to serve as input for the project management in developing the project.

***Data collection***

The data material for this article consists of individual interviews and focus groups with the project staff (SSIA and SPES officials), their project leaders and managers, and representatives from the steering group of the project. In total, 17 focus groups and 28 individual interviews were carried out. Focus groups with the project staff consisted of the same participants, albeit in different constellations. Interviews were done twice with the same person in 5 cases, at different time points. The material was collected throughout the project, from 2012 to 2014. The timeline for the data collection is described in Figure 1.

(Place Figure 1 about here.)

Each round of interviews and focus groups focused on the current situation in the project, along with specific themes. In the first focus group with the staff, the focus was on their expectations of the project; their previous work experience; and perspectives on central concepts (such as work ability). In subsequent focus groups with the staff, focus was on organizational conditions for performing their work; on the use of specific methodologies (primarily MI); case examples; and on any other topics that they felt a need to discuss. In the focus groups with case examples, officials were asked to bring (anonymous) cases where they reached good and bad results, which was used for discussions about which factors that influenced these outcomes. Focus groups consisted of about 10 people per group, most commonly divided into the work groups from the different offices, but on two occasions in mixed groups (the initial focus groups, and the focus groups focusing on case examples). At the closure of the project, individual interviews were carried out with 10 representatives from the staff, focusing on their retrospective perspective of the project, and on their own professional development. Focus groups and interviews with managers and project leaders were focused on the development of the project, and on their managerial roles and strategies. Interviews with steering group representatives and the funding agency were primarily focused on the development of the project with special attention to their perspectives on utilization and implementation of project results. The focus groups were led by the first author, assisted by the second and in some cases the fourth author. The first author carried out the interviews. All interviews and focus groups were carried out face-to-face.

***Analysis***

All focus groups and interviews were transcribed verbatim. The material was analyzed through a qualitative content analysis [[13](#_ENREF_13)]. This was done in several steps, where preliminary analyses were carried out after each wave of interviews and focus groups, for the continuous feedback reports to the project. At this stage, the first, second and fourth author read all transcripts and divided the first coding of the material between them, followed by discussions to reach consensus on the final codes. After the project ended, these analyses were used as a starting point for a more selective analysis, were material related to the research questions of the process evaluation was identified and analyzed again. This material was organized into categories and sub-categories, using an inductive approach. The first author drafted an initial coding that was discussed with the other authors until consensus was reached. In this process, two broad categories were identified: 1) conditions for developing and managing cooperation, and 2) experiences of cooperative casework. These categories comprised several sub-categories. The first included three sub-categories: establishing a cooperative project; balancing development and production requirements; and feedback, support and conditions for learning. The second included two sub-categories: holistic and flexible casework; and the use of and fidelity to the principles of MI. Illustrative quotes were selected and translated into English.

In the analysis, the inductively derived codes were related to theories, where social capital was identified as a concept with explanatory value to the experiences of cooperative work based on the focus on trust in the results, which is a key issue in social capital theory [[14](#_ENREF_14)], while implementation issues were related to previous literature on the challenges of implementing projects [[15](#_ENREF_15)].

**Results**

The staff working in the project perceived it both as a positive and a negative experience; the positive aspects were associated with case work in close cooperation between authorities; the negative aspects were associated with the organization and management of the project. Managers, who also expressed critique regarding the design and development of the project, largely mirrored these perceptions.

***Conditions for developing and managing cooperation***

The first category is related to the challenges in designing and running a large cooperation project.

*Establishing a cooperative project*

The design of the Dirigo project was first described in the application for funding to the European Social Fund (ESF). The goals of the project were formulated on a theoretical basis about what could be expected with regard to the intended target groups. However, the process for recruitment of participants was difficult and the final group of participants had more complicated problems than first anticipated. Further, in retrospect, managers could see how some of the elements originally meant to be included were not possible to get in place, e.g. cooperation with municipal and health care services, and having close ties to employers. Managers also expressed how there were many challenges related to setting up interorganizational workspaces, where they needed to merge different regulations about how offices were designed, and where the absence of a common administrative system was troublesome.

One effect of the recruitment problems was that it took a long time before the first participants were included. The consequence of this was that the staff spent their first months engaging in educational activities and discussions. This spurred the staff to participate in development work. However, the results of this were not put into practice due to later budget restrictions, which was experienced as disappointing.

If you give the staff these fictive mandates, of course, it will be… It creates disappointment and ruins their trust in the management when we give them these tasks and don’t take care of the results. (Project leader)

During the first year, the steering group changed the goals of the project. These were not changed to fit the actual participants, but towards a higher success rate (see Table 1). The goals of the project did not match what could be expected from the actual group of participants, at least not within the time frame of the project. One effect of this was that the staff did not consider the new goals to be relevant, and they therefore did not attempt to achieve them. Instead, officials formulated their own goals based on what they thought would be reasonable to expect.

We didn’t even accept the new goals, we just laughed. So as far as I’m concerned, they don’t exist. […] My goal has been that these people that I work with will get closer [to work or studies]. (Staff)

There were also problems related to staffing on the managerial level, where the project had three managers over the two years. Further, several of the staff left the project. In the focus groups with the staff, a recurrent issue was the perceived lack of structure and clarity in the design of the project, where the staff complained about getting too little information about decisions and changes. The recruitment problems and the instable managerial situation contributed to a feeling among the staff that the project was not designed well, and that there were flaws both regarding its organization and its goals. The staff also perceived lacking communication in relation to the management level. The relationship between managers and staff was therefore strained; distrust and dissatisfaction was continuously expressed in focus groups and interviews.

The ambiguities regarding goals and resources and the issues related to staffing and recruitment of participants contributed to dissatisfied and distrustful staff members. The project management underestimated the practical problems involved with setting up the project, where problems had to be solved ad hoc without the staff being informed. The relationships among the staff did however seem more positive, where several officials expressed being very satisfied with the work group. Some differences could be traced among the different offices, where especially one of them seemed to have had a strong group feeling. In this office, the manager was also considered to be very supporting, in contrast to how the rest of the management was perceived. In the other offices, positive relationships were more limited to the pairs of officials working together.

*Balancing development and production requirements*

One problem during the project was that the funding structure probed a strong focus on quantitative measures. The funding was tied to the number of hours that participants were engaged in activities, which implied that much time was spent on finding activities and reporting these. A possible perverse effect of the funding system, at least theoretically, was that it could be more beneficial for the project in terms of funding to keep a participant in activities for a long time, rather than to offer a shorter intervention that helped them to find a job. A consequence of this system was that attention to quantity rather than content was promoted.

It has been so much administration […], “how are we going to achieve all those hours?”, not “how should we best work with this person?”. You can take that temporarily […] but then you need to think about the next step. How do we continue to work with this in terms of quality? (Staff)

According to a steering group representative, the project could have managed the funding system by trusting that the methods would provide a good result, and focus less on the number of hours spent on activities.

We have said that we cannot do this type of hunt for hours; we have to work with what people actually need. And we think that when we identify what people need and understand what tools we have at our disposal, then the hours will come naturally. (Steering group)

The project managers, however, did the opposite – starting with securing the number of hours, and then using the remaining time for developing the methods. The result of this, according to the staff, was that time for development was scarce.

The recruitment problems and the many changes in staffing and organization meant that the project was heavily occupied with trying to survive rather than developing the actual work. The staff experienced too much steering in relation to production requirements, and too little in relation to development, which was left to the staff to work out among themselves.

*Feedback, support and conditions for learning*

Since the idea with the project was to develop new methods through cooperation, there was an expectation from the staff that they were to be given time for discussion, to receive feedback and to be supported by the management in developing their work. The many problems related to establishing the project took much time and energy from the development of methods in the client work and the staff continuously noted how they received too little feedback and support in their everyday practice, and that they were left to support each other. Generally, they perceived the work they did as rewarding and developing, and they reported using each other in the pairs as resources for developing their work; on a work group level, however, this was not discussed sufficiently. The staff requested more attention from the management to these issues, but perceived the management as too occupied with securing that production requirements were met. Halfway through the project, the then newly recruited project manager introduced joint meetings between the offices to focus on developmental issues, but the staff perceived this as being too little and too late.

I have felt sorry for [the project manager] in a way, because she has been standing there trying to achieve something, and I just felt “yeah, great, but a year too late”. […] Here’s a bunch of people who looks so dead tired and grumpy, including myself, and it’s Monday morning and we’re pretty despondent. (Staff)

Further, it took time until the staff received proper coaching. After about a year, coaching in insurance medicine was offered to the SSIA officials – a service offered in regular practice to support officials in managing their cases that the project staff had lacked. Once introduced, this was perceived as helpful. SPES officials did not receive any comparable coaching, but could participate in sessions with the insurance medicine tutor. Coaching in specific methods, especially MI, was also lacking throughout the project. Further, the managers were not trained in MI, which impeded their abilities to support the staff in applying of the method.

In summary, the first category shows how the establishment of Dirigo had several flaws that resulted in a suboptimal development. The staff perceived a lack of both feedback and support in their work with participants, and there were several ambiguities regarding project goals, staffing and the management structure. The staff was largely left to provide feedback through peer support, where little managerial attention was given to organizing such feedback. The conditions for learning new methods and developing interorganizational casework were therefore not optimal.

***Experiences of cooperative casework***

The second category considers the experiences of cooperative casework within the project. This was primarily discussed by the staff, and was mentioned to a lesser extent by the managers.

*Holistic and flexible casework*

The staff experienced their work with participants in the project as very positive and rewarding, and they reported that the participants experienced the intervention as helpful. The officials were in agreement that they were able to apply a holistic perspective to the participants’ situation and engage in tailored motivational and change-oriented work based on this information, thereby involving the participants on their own terms.

The staff identified several specific factors that facilitated this holistic approach:

- continuity and flexibility in the casework;
- using MI;
- reduced case load compared to regular practice in the authorities;
- shared responsibility for cases between the authorities;
- sharing the same workspaces.

These factors combined, according to the staff, resulted in a trustful relationship between the participant and the officials, leading to realistic recovery and rehabilitation plans. As a result of the shared responsibility for the cases, the participants gained access to resources and interventions from both the SSIA and the SPES, which was a unique feature for this group who, in regular practice, had limited or no access to the more activation-oriented measures offered by the SPES. When participating in the project, it was possible to receive compensation from one authority (sickness or disability benefits) and rehabilitation measures from another. Thus, a crucial ingredient of the casework was the availability of activities and rehabilitation measures offered in regular practice; the additional factors in the project were flexible case management and cooperation between officials from different authorities.

It’s a new role, you put this insurance official role aside. I look to the person, the participant as a person we work with, not what authority we come from. […] We’re working to gain their trust, and to be able to work differently compared to regular practice. (Staff)

The close cooperation between the SSIA and the SPES officials fostered a flexible application of procedures within the authorities, since these were often not perceived as relevant when working in an inter-organizational milieu. This was perceived as positive since it reduced the influence of organizational boundaries in finding solutions to client needs. Shared workspaces also allowed for fast and flexible communication between the authorities.

In cases where positive results were not reached, explanations were given on an individual level (e.g., medical reasons or lacking motivation), an organizational level (limited access to rehabilitative measures) and a societal level (lack of available jobs).

*Use of and fidelity to the principles of MI*

MI was a guiding method for the officials when working with participants. The staff was offered four days of training in the method, and although follow-up of the training was perceived as lacking or carried out haphazardly, the officials seemed to consider themselves as able to apply MI principles reasonably well. The officials emphasized how it is relatively easy to understand the basic principles, but that mastering it as a method takes a lot of training. The officials were able to develop their skills in their casework, but they lacked a structure for reflection while developing their practical knowledge.

Most officials expressed positive experiences of applying MI in the casework, given that they had the time for engaging with participants in a more intense and flexible way compared to regular practice. The method was applied differently on a case-by-case basis; sometimes, however, MI was not used at all.

I’ve got a new tool to use, but that doesn’t mean I use it every time, it depends on the situation. It’s like any other tool; I mean, if I need a hammer, I don’t use a screwdriver. (Staff)

In terms of fidelity to the principles of MI, the general principles of the MI “spirit” seemed to be applied generally, especially with regard to letting the participants set the agenda and define the needs for change. In some cases, some of the more specific tools were applied, depending on the official’s level of competence. Most officials claimed to use MI freely, more as an approach than as a method, and it was primarily those officials who received extra training (a few officials participated in another project in which they were given this opportunity) that appeared to use it on a more advanced level. It was apparent how the staff considered working in a project with reduced caseload facilitated their use of MI; most of the staff did not expect to be able to use it once the project ended, due to the heavy caseload in regular practice.

In summary, the second category illustrates how the staff in the project managed to carry out cooperative casework that significantly differed from the routines in regular practice, even in the face of many organizational obstacles. The many problems related to establishing the project had a negative effect on the possibilities for developing and carrying out the case work.

**Discussion**

In this section, the establishment and development of the project will be discussed in relation to two broad themes: trust and social capital in cooperative work; and implementation challenges in interorganizational cooperation.

***Trust and social capital in cooperative work***

Despite the many problems with regard to the organization, recruitment, staffing and management of the project, the staff reported the work as rewarding and positive in relation to the participants and in relation to their colleagues. The results thereby points to the strength of close, tailored cooperation, where time and attention to building relationships with participants were perceived as crucial elements. Developing a working alliance with the participant is one of the key aspects of the MI method [[16](#_ENREF_16)], which seems to have been possible to achieve within the context of the project.

The staff reported a high amount of trust between participants and staff, as well as between staff members. One way of conceptualizing this trust is through social capital, where these results illustrate how cooperative work promoted the development of social capital both within the work group and in relation to participants in the casework. Social capital may be defined in different ways and distinctions between different varieties have been suggested [[14](#_ENREF_14), [17](#_ENREF_17)].

Bonding social capital refers to trusting and co-operative relations between members of a network who see themselves as being similar, in terms of their shared social identity. Bridging social capital, by contrast, comprises relations of respect and mutuality between people who know that they are not alike in some socio-demographic (or social identity) sense. […]We would define linking social capital as norms of respect and networks of trusting relationships between people who are interacting across explicit, formal or institutionalized power or authority gradients in society. [[14, p. 654-655](#_ENREF_14)]

This distinction between bonding, bridging and linking social capital is useful for categorizing different relationships. In the project, bonding social capital was apparent in the cases where officials developed a group feeling. The cooperation and shared responsibility between officials from different authorities in this study promoted an increased bridging social capital. This in turn facilitated the case management by fostering a mutual understanding and access to resources and rehabilitation measures from more than one authority. In relation to the participants, the project promoted linking social capital through the use of MI and the flexibility in case management, where the staff perceived to get a better relationship to their clients compared to regular practice.

Although being used to working bureaucratically in their ordinary practice, officials participating in cooperative work take on a broader perspective on managing more complex situations [[7](#_ENREF_7), [18](#_ENREF_18)]. Thus, it seems that cooperative work tends to go hand in hand with a holistic and solution-based approach to peoples’ problems. Organizing interorganizational cooperation therefore seems to imply organizing for officials’ discretion, which makes its popularity among the officials understandable. A high level of discretion could imply better decisions due to the increasing possibility of taking individual conditions into consideration. However, increased discretion through cooperation could likewise imply the opposite, since it also involves a risk of arbitrary decisions. On the other hand, as Lipsky [[19](#_ENREF_19)] shows, increasing governance does not automatically mean that decisions are made in compliance with policy, which means that the risk of arbitrary decisions is constantly present.

***Implementation challenges in interorganizational cooperation***

Dirigo was a time-limited project primarily financed through external funds, and as is common for such projects, implementation of the project methods into regular practice was an issue. The project could provide resources that are not possible to muster in regular practice, e.g., reduced caseload and discretion to test new approaches. Therefore it is important to relate the results and experiences from the project to the conditions in the respective organizations when considering the applicability and possibility of implementing principles and methods applied in the project. Further, as in many projects, a project logic dominated the work (i.e., planning and carrying out activities, and reporting results), implying a focus on administration and control [[20](#_ENREF_20)]. Demands for short-term results tend to overpower the ambitions to develop sustainable long-term effects, which was clearly the case in this project.

The results indicate that a combination of the unique features of the project (MI, reduced caseload, flexibility and close cooperation) and measures offered in regular practice (e.g., subsidized employments, rehabilitation measures) was positive for the work with participants. To achieve similar results in regular practice, the staff would need to be offered similar discretion and similar resources. However, regular practice is not organized to facilitate close cooperation: work routines limit the interactions between the SSIA and the SPES to specific time points, and officials are placed in different locations.

It may also be argued that the authorities involved, being state authorities whose practices are largely directed through political decisions, have a limited readiness for change [[15](#_ENREF_15)], especially regarding the uptake of methodologies that require organizational restructuring. In the project, organizational factors severely impeded the establishing of the project and the possibilities for the project to reach its goals (e.g., managerial problems, staffing, and difficulties in managing administration and documentation without a joint system). In this case, the managers tried to find local solutions to problems related to organizational structure and procedures decided on a state level. Implementing the project results would require new cooperative offices to be established where officials from the two authorities could work together. While there are no legal obstacles to doing so, the interviews with the steering group did not suggest any such plans. Hence, the problems related to implementation are due to structural problems as well as to individuals who are resistant to acting on the results in changing regular practice. The largely informal cooperation structure developed in the project would therefore need to be formalized in order to facilitate a more systematic implementation, and to prevent that cooperation is developed locally on a trial-and-error basis.

Another potential long-term effect of the project could be the uptake of MI, which was considered a positive method for meeting participants. The use of MI was often contrasted with the conditions in regular practice, and the staff did not expect to be able to use the method once the project ended. Hence, the project illustrates how organizational conditions matter for whether a method may be applied with fidelity. The uptake of MI in regular practice is therefore an implementation issue that requires not just education, but adjustments in terms of feedback and support systems in the organizations.

The experiences from the Dirigo project mirrors the conclusions from previous studies on cooperation, e.g., Huxham’s conceptualization of collaborative advantage (the potential for synergies of working collaboratively) and collaborative inertia (the often disappointing output in reality). As she notes, the results of collaboration is often not seen on the short term, and “stories of pain and hard grind are often integral to the success achieved” [[21, p. 403](#_ENREF_21)]. The inertia described by the respondents in this study is related to the time-limited project form, and to the expectation of not being able to use the experiences and methodologies (i.e., the collaborative advantage) developed when the project is ended. This, unfortunately, seems like a well-grounded concern.

***Methodological considerations***

The results reported here are parts of a larger study, where the participants’ experiences of the Dirigo project will be presented in a forthcoming article. As a consequence, the perspectives of the participants are not reported here. Further, the generalizability of the study is limited to similar cooperative projects between authorities, which means that the results should be interpreted with respect to the specific context. The study is primarily based on focus groups and interviews, while no observations were made of the actual work in the project. The material is however large enough to allow an analysis of the project development from the perspective of the staff. The trustworthiness of the study was increased by the continuous feedback reports written throughout the project, which was a basis for a dialogue with the project staff and management about the emerging results.

**Conclusions**

The establishment and development of the Dirigo project illustrates several issues related to the design, organization and management of cooperative work, issues that may have contributed to the project failing to reach its goals. The process evaluation shows how several factors contributed to the difficulties, e.g. recruitment of participants, the funding structure, and staffing problems on the managerial level, which resulted in a suboptimal development, and distrust between managers and officials. The many organizational problems surrounding the project, combined with the relatively large differences in approaches between the project and regular practice, also obstructed implementation of the methods used in the project. It may further be argued that the authorities involved have a limited readiness for change, especially when the uptake of new methodologies requires organizational restructuring.

The evaluation does however point to positive effects of close cooperation, from the perspective of the officials. Shared responsibility between officials from different authorities promoted an increased bonding and bridging social capital between officials, which in turn facilitated the case management by fostering a mutual understanding and access to resources and rehabilitation measures from more than one authority. In relation to the participants, the project seems to have promoted linking social capital through the use of MI and the flexibility in case management.

Taken together, the results of this study illustrate the tension between ambitions to develop practice, and the organizational realities that such ambitions meet when implemented. These complexities of and challenges in translating ideas into practice are important to consider when planning and implementing interventions, especially in an interorganizational setting.

***List of abbreviations***

ESF: European Social Fund

MI: Motivational Interviewing

SPES: Swedish Public Employment Service

SSIA: Swedish Social Insurance Agency

**Declarations**

***Ethics approval and consent to participate***

The regional research ethics committee in Linköping approved of the study (Dnr 2012/115-31). All participants gave informed verbal consent to participation in the study. Written consent was not considered necessary, since no personal information was collected; the interviews focused solely on the respondents’ professional work. The ethics committee approved of this procedure.

***Consent for publication***

Not applicable.

***Availability of data and materials***

Data is available from the first author on request.

***Competing interests***

The authors declare no conflicts of interest.

***Funding***

The project was financed by the ESF, the SSIA, the SPES, and by the participating municipalities. The funding organizations had no role in the planning, execution or analyses of the study.

***Authors' contributions***

CS and IA designed the study, and CS, ÅA and KL carried out the data collection. Preliminary analyses were carried out by all authors. CS wrote most of the manuscript; ÅA, KL and IA contributed with comments. All authors read and approved the final manuscript.

***Acknowledgements***

The authors would like to acknowledge Kerstin Ekberg for contributing to the design of the study.

**References**

1. Hultberg E-L: **Co-financed collaboration between welfare services: Effects on staff and patients with musculoskeletal disorders**. Göteborg: Göteborg University; 2005.

2. Martin MHT, Nielsen MBD, Madsen IEH, Petersen SMA, Lange T, Rugulies R: **Effectiveness of a Coordinated and Tailored Return-to-Work Intervention for Sickness Absence Beneficiaries with Mental Health Problems**. *Journal of Occupational Rehabilitation* 2013, **23**(4):621-630.

3. Bültmann U, Sherson D, Olsen J, Hansen CL, Lund T, Kilsgaard J: **Coordinated and tailored work rehabilitation: A randomized controlled trial with economic evaluation undertaken with workers on sick leave due to musculoskeletal disorders**. *Journal of Occupational Rehabilitation* 2009, **19**(1):81-93.

4. Franche R-L, Cullen K, Clarke J, Irvin E, Sinclair S, Frank J, Cole D, Dacombe J, Guzman J, Hogg-Johnson S *et al*: **Workplace-Based Return-to-Work Interventions: A Systematic Review of the Quantitative Literature**. *Journal of Occupational Rehabilitation* 2005, **15**(4):607-631.

5. Jakobsson B, Ekholm J, Bergroth A, Ekholm KS: **Improved employment rates after multiprofessional cross-sector cooperation in vocational rehabilitation: A 6-year follow-up with comparison groups**. *International Journal of Rehabilitation Research* 2010, **33**(1):72-80.

6. Kärrholm J: **Co-operation among rehabilitation actors for returning to working life**. Stockholm: Karolinska Institutet; 2007.

7. Ståhl C: **In Cooperation We Trust: Interorganizational Cooperation in Return-to-Work and Labour Market Reintegration**. Linköping: Linköping University; 2010.

8. Fridolf M: **Integrering som lokal organiseringsprocess mellan professionella, chefer och politiker**. Göteborg: Göteborg University; 2002.

9. **Samverkan mellan Arbetsförmedlingen och Försäkringskassan under 2000-talet (Cooperation between the Social Insurance Agency and the Public Employment Service in the 2000's)**. In*.*, vol. 2010:04. Stockholm: Parlamentariska socialförsäkringsutredningen, Statens offentliga utredningar; 2010.

10. Hettema JE, Steele JM, Miller WR: **Motivational Interviewing**. *Annual Review of Clinical Psychology* 2005, **1**:91-111.

11. Miller WR, Rollnick S: **Motivational interviewing: Helping people change**, 3 edn. New York: Guilford Press; 2013.

12. Svensson L, Brulin G, Jansson S, Sjöberg K (eds.): **Learning through ongoing evaluation**. Lund: Studentlitteratur; 2009.

13. Patton MQ: **Qualitative research and evaluation methods**, 3 edn. London: Sage; 2002.

14. Szreter S, Woolcock M: **Health by association? Social capital, social theory, and the political economy of public health**. *Int J Epidemiol* 2004, **33**(4):650-667.

15. Damschroder LJ, Aron DC, Keith RE, Kirsh SR, Alexander JA, Lowery JC: **Fostering implementation of health services research findings into practice: a consolidated framework for advancing implementation science**. *Implementation Science* 2009, **4**(1):art. no. 50.

16. Luciano A, Drake RE, Bond GR, Becker DR, Carpenter-Song E, Lord S, Swarbrick P, Swanson SJ: **Evidence-based supported employment for people with severe mental illness: Past, current, and future research**. *Journal of Vocational Rehabilitation* 2014, **40**(1):1-13.

17. Putnam RD: **Bowling Alone: The Collapse and Revival of American Community**. New York: Simon & Schuster; 2000.

18. Ståhl C, Svensson T, Petersson G, Ekberg K: **A Matter of Trust? A Study of Coordination of Swedish Stakeholders in Return-to-Work**. *Journal of Occupational Rehabilitation* 2010, **20**(3):299-310.

19. Lipsky M: **Street-Level Bureaucracy: Dilemmas of the Individual in Public Services**. New York: Russell Sage Foundation; 1980.

20. Svensson L, Brulin G: **Effects of programmes and projects**. In: *Capturing effects of projects and programmes.* edn. Edited by Svensson L, Brulin G, Jansson S, Sjöberg K. Lund: Studentlitteratur; 2013: 15-34.

21. Huxham C: **Theorizing collaboration practice**. *Public Management Review* 2003, **5**(3):401-423.

**Figure captions**

Figure 1: Timeline for data collection and feedback reports to the project.

**Additional files**

Guides for interviews and focus groups are presented as additional files. These include the following guides with corresponding file names:

- 2012 guide for focus group with managers
- 2012 guide for spring focus groups with staff
- 2012 guide for fall focus groups with staff
- 2012 interview guide for departing manager
- 2012 interview guide for managers
- 2012 interview guide for steering group
- 2013 guide for spring focus groups with staff
- 2013 guide for fall focus groups with staff
- 2013 interview guide for managers
- 2014 interview guide for funders
- 2014 interview guide for managers
- 2014 interview guide for staff
- 2014 interview guide for steering group
