# Supplementary material for: Process evaluation of an interorganizational cooperation initiative in vocational rehabilitation: the Dirigo project
Source: BMC Public Health. 2017 May 11;17:431. doi: 10.1186/s12889-017-4357-x (PMC5426082; doi:10.1186/s12889-017-4357-x)
Supplement: Additional file 1: — Guides for interviews and focus groups. (ZIP 240 kb) [file 12889_2017_4357_MOESM1_ESM.zip › 2012 interview guide for steering groupR3.doc]

Interview guide, steering group, 2012

- What is your background, and how did you get to be involved in this project?
  - Were you involved in developing the project from the beginning?
- How did you choose the methods to be used in the project?
- How will the methods be applied?
- Will they work in teams, pairs, or something else?
- Is there a strategy for connecting with employers?
- How do you think the project will affect your organization’s way of working?
  - Will it affect structures, routines, working methods?
- There are many things in the project that is not on place in the regular organizations, do you think that this will make the project methods difficult to implement?
  - How will you deal with this?
- In measuring results and effects from the project, what is your perspective on what any potential differences compared to regular practice will depend on? What are we measuring?
- Anything else you would like to discuss?
