# Supplementary material for: Process evaluation of an interorganizational cooperation initiative in vocational rehabilitation: the Dirigo project
Source: BMC Public Health. 2017 May 11;17:431. doi: 10.1186/s12889-017-4357-x (PMC5426082; doi:10.1186/s12889-017-4357-x)
Supplement: Additional file 1: — Guides for interviews and focus groups. (ZIP 240 kb) [file 12889_2017_4357_MOESM1_ESM.zip › 2014 interview for guide steering groupR3.doc]

**Interview guide, steering group 2014**

- What have you learned professionally during this project?
- What expectations did you have on the project?
- The project changed during its course, what were the reasons for this?
- Did you have to refrain from doing things you had hoped to do in the project?
- What, in your perspective, has the project achieved?
- The project had some star-up problems, what are your perceptions of that today?
- How was the relationships between the steering level, the management and the staff?
- How has the regular organizations prepared for having their employees back? Do they get any particular positions or tasks? Are they involved in this?
- How did the project methods relate to work routines in the regular organizations?
- How involved are you personally in the closing of the project?
- Is there anything else we should consider when writing our final report on the project?
